# Special Section on Digital Health for Precision in Prevention: Notable Papers that Leverage Informatics Approaches to Support Precision Prevention Efforts in Health Systems

**DOI:** 10.1055/s-0044-1800721

**Published:** 2025-04-08

**Authors:** Brian E. Dixon, John H. Holmes, Section Editors of the Special Section on Inclusive Digital Health

**Affiliations:** 1 Department of Health Policy & Management, Richard M. Fairbanks School of Public Health, Indiana University, Indianapolis, IN, USA; 2 Center for Biomedical Informatics, Regenstrief Institute, Indianapolis, IN, USA; 3 Department of Biostatistics, Epidemiology, and Informatics, University of Pennsylvania Perelman School of Medicine, Philadelphia, PA, USA; 4 Institute for Biomedical Informatics, University of Pennsylvania Perelman School of Medicine, Philadelphia, PA, USA

**Keywords:** Public Health Informatics, Primary Prevention, Secondary Prevention, Precision Medicine, Digital Health

## Abstract

**Objective** : To identify notable research contributions relevant to digital health applications for precision prevention published in 2023.

**Methods** : An extensive search was conducted to identify peer-reviewed articles published in 2023 that examined ways that informatics approaches and digital health applications could facilitate precision prevention. The selection process comprised three steps: 1) candidate best papers were first selected by the two section editors; 2) a diverse, international group of external informatics subject matter experts reviewed each candidate best paper; and 3) the final selection of four best papers was conducted by the editorial committee of the Yearbook. The section editors attempted to balance selection by authors' global region and areas with clinical medicine and public health.

**Results** : Selected best papers represent studies that advanced knowledge surrounding the use of digital health applications to facilitate precision prevention. In general, papers identified in the search fell into one of the following categories: 1) applications in precision nutrition; 2) applications in precision medicine; and 3) applications in precision public health. The best papers spanned several disease targets, including Alzheimer's disease, HIV, and COVID-19. Several candidate papers sought to improve prediction of disease onset, whereas others focused on predicting response to interventions.

**Conclusion** : Although the selected papers are notable, significant work is needed to realize the full potential for precision prevention using digital health. Current data and applications only scratch the surface of the potential that information technologies can bring to support primary and secondary prevention in support of health and well-being for all populations globally.

## 1. Introduction

Building off the reviews by Pearson *et al.* [ 1 ] and Gallego *et al.* [ 2 ], the survey paper commissioned for the 2024 Yearbook of Medical Informatics, we define *precision prevention* as “ *the use of biologic, behavioral, socioeconomic, and epidemiologic data to inform the right intervention at the right intensity in the right community at the right time, in order to prevent or reduce illness and improve health* ”. The term grew out of the work started two decades ago in *precision medicine* , which calls for individualizing patient treatments based on genetic and biomedical markers, and later gave rise to the notion of *precision public health* , which emphasizes tailoring of community-level interventions to specific populations that may benefit most from a given intervention.

An illustration of precision prevention can be found in Lim *et al.* [ 3 ], which examined the evidence on interventions for pregnant women with respect to preventing gestational diabetes mellitus (GDM). The scientists examined more than 10,000 studies to find evidence on the use of pregnant persons' biological, genetic, and lifestyle characteristics to predict response to a given intervention that aimed to prevent GDM. The researchers found that combined diet and physical activity worked best for pregnant persons without polycystic ovary syndrome (PCOS) versus those with PCOS. Patients with PCOS responded better to metformin interventions. Understanding which subpopulations respond better to which interventions can help clinicians and public health programs better prevent onset of disease or support secondary prevention of morbidity associated with disease.

Despite a decade of work to develop precision prevention techniques and studies on precision public health approaches, little is known about the role of informatics in supporting precision prevention. In theory, digital health applications and informatics approaches are critical to ensure clinical and public health organizations collect, manage, and analyze the broad landscape of data ( *e.g.* , clinical, demographic, behavioral, environmental, social, educational) necessary to deliver precise interventions to populations. It is precisely for this reason that the International Medical Informatics Association (IMIA) Yearbook of Medical Informatics Selection Committee chose “Digital Health for Precision in Prevention” as this year's theme. The special section of the Yearbook focuses on calling out recent, high-quality publications that examine and/or advance our understanding of informatics in facilitating precision prevention.

## 2. Methods

The primary author (BED) performed a literature search using PubMed in January 2024. The query was developed to broadly search biomedical journals for articles pertaining to digital health applications relevant to precision prevention. Both controlled vocabulary terms ( *e.g.* , Medical Subject Headings) and text words were used. Because precision prevention is relevant to many biomedical domains, we explicitly included terms like “precision nutrition” and “precision public health” in addition to “precision prevention”. Also, because many precision medicine approaches involve the use of advanced analytics, including artificial intelligence in order to predict who might be at risk for a disease or outcome, we included “artificial intelligence” as an informatics search term. We employed Boolean logic to identify articles published in English language between January 1, 2023 and December 31, 2023, that contained at least one information science term *and* one precision prevention related term. The full query is included as Appendix A.

Information retrieval yielded 126 candidate articles. Using Covidence systematic review software (Veritas Health Innovation, Melbourne, Australia 1 ), the editors performed initial screening of titles and abstracts. Screening removed 70 studies that did not pertain to the theme. Both editors reviewed the remaining articles and categorized them into three groups (accept, discuss, and discard) based on their innovativeness, scientific and/or practical impact, and methodological quality. Reasons for exclusion included descriptive review articles or commentary/editorial; published prior to 2023; lacking peer review (published as preprint); or the contribution of informatics methods or approaches was unclear.

This process yielded 11 articles as candidate best papers [ 4 5 6 7 8 9 10 11 12 13 14 ]. In accordance with the IMIA Yearbook selection process, the candidate best papers were further evaluated by the two section editors, the chief editor of the section, and by additional external reviewers (at least four reviewers per paper) with expertise in medical and/or public health informatics.

## 3. Results

The search yielded a small corpus of papers related to precision prevention involving information systems. Several of these papers were commentaries or perspective articles that called for action, encouraging or outlining how digital health could be used to support precision prevention efforts [ 4 5 6 7 ]. However, these articles were excluded from consideration as best papers. Instead, our focus was on those papers that detailed the development, implementation, and/or evaluation of a digital health solution that aimed to support precision prevention. Although candidate papers did not have to propose a novel digital health application, they had to be relevant to biomedical informatics. Some excluded papers, for example, involved clinical interventions for precision prevention that did not pertain to digital health or involve information systems.

Candidate papers relevant to informatics could be categorized into one of three groups: 1) *precision* *nutrition applications* , such as the use of machine learning to predict response to a dietary fiber intervention based on metabolism and/or gut microbiome [ 15 ]; 2) *precision medicine* , in which the use of machine learning or artificial intelligence (AI) is used to predict therapeutic response to treatments; or 3) *precision public health* , applications in which analytics are utilized to predict response to social or behavioral interventions in order to prevent disease onset or result in harm reduction ( *e.g.* , smoke cessation).

Most candidate papers could be categorized as precision nutrition applications. While many used machine learning or AI approaches to predict response to interventions or outcomes given genetic risk profiles, several papers pertained to infrastructure needed to advance precision nutrition approaches. One paper, for example, described the development of a database of food-drug interactions that could be used to power a clinical decision support system designed to detect and alert patients to potential interactions that might prevent effectiveness of therapeutics prescribed by their clinicians [ 8 ]. Another paper described a database designed for use with digital health applications ( *e.g.* , wearables) that could provide customized nutrition and lifestyle guidelines to patients [ 9 ]. These applications make us excited about the power of AI and machine learning to help patients make healthy choices when eating and create customized nutrition plans to optimize health, including weight loss and muscle development, in the future.

The other set of papers that excited us were those focused on precision public health applications. Some of these papers were clinically oriented, such as the risk communications trial from Guan *et al.* [ 10 ]. In this trial of women at average risk for breast cancer, some women received customized risk communications that provided alternatives to annual mammograms. These alternatives included delaying screening or reducing the frequency of screening in alignment with recommendations from the United States Preventative Services Task Force. Armed with genetic, behavioral, and mammography risk information, women in the intervention arm were more willing to delay or reduce frequency of screening. The trial demonstrated that customized risk information can be useful in helping patients choose appropriate screenings in addition to treatments and lifestyle interventions. More evidence on precision public health applications using dashboards, smart devices, SMS, patient kiosks, etc. would be useful to help populations increase awareness of their risks and the benefits of more tailored interventions designed to keep them healthy.

The papers selected by the section editors in consultation with the editorial board as best papers are summarized in Table 1 [ 11 12 13 14 ]. Final selection was based on these criteria: 1) reviewer ratings and comments; 2) equity across authors' nation and world regions; and 3) content balance across disciplines in medicine and public health. Some disciplines had more candidate papers than others, and the editors felt that all disciplines should be represented in the special section. A content summary of the selected best papers can be found in Appendix B of this synopsis.

**Table 1.** Number of papers collected in the PubMed database from 2013 to 2023 for each country based on the first author's affiliation country.

Notable Papers Published in 2023 focused on Digital Health for Precision Prevention
Andrade AQ, Calabretto JP, Pratt NL, Kalisch-Ellett LM, Le Blanc VT, Roughead EE. Precision public-health intervention for care coordination: a real-world study. Br J Gen Pract. 2023;73(728):e220-e30.
Hwang U, Kim SW, Jung D, Kim S, Lee H, Seo SW, et al. Real-world prediction of preclinical Alzheimer's disease with a deep generative model. Artif Intell Med. 2023;144:102654.
Lee S, Kim S, Yoon DS, Park JS, Woo H, Lee D, et al. Sample-to-answer platform for the clinical evaluation of COVID-19 using a deep learning-assisted smartphone-based assay. Nat Commun. 2023;14(1):2361.
Wang B, Liu F, Deveaux L, Ash A, Gerber B, Allison J, et al. Predicting Adolescent Intervention Non-responsiveness for Precision HIV Prevention Using Machine Learning. AIDS and behavior. 2023;27(5):1392-402.

## 4. Conclusions

The best papers on digital health for precision prevention in 2023 represent a fraction of the strong scientific articles relevant to this topic published before and after this synopsis. Given rising rates of noncommunicable morbidity and mortality following the COVID-19 pandemic, precision prevention is in the forefront of the work we must do in digital health. Continued research and development is necessary to implement digital health tools that support precision prevention for all persons across the globe.

## Footnotes

1
https://www.covidence.org/
